# Repeat biopsy versus initial biopsy in terms of complication risk factors and clinical outcomes for patients with non-small cell lung cancer: a comparative study of 113 CT-guided needle biopsy of lung lesions

**DOI:** 10.3389/fonc.2024.1367603

**Published:** 2024-05-13

**Authors:** Yangyang Wang, Yongyuan Zhang, Nana Ren, Fangting Li, Lin Lu, Xin Zhao, Zhigang Zhou, Mengyu Gao, Meng Wang

**Affiliations:** ^1^Department of Medical Imaging, The Third Affiliated Hospital of Zhengzhou University, Zhengzhou, China; ^2^Department of Medical Imaging, Zhengzhou People’s Hospital, Zhengzhou, China; ^3^Department of Medical Imaging, The First Affiliated Hospital of Zhengzhou University, Zhengzhou, China

**Keywords:** non-small cell lung cancer, rebiopsy, computed tomography-guided, tyrosine kinase inhibitors, acquired drug resistance, chemotherapy

## Abstract

**Objectives:**

The safety and feasibility of repeat biopsy after systemic treatment for non-small cell lung cancer have received extensive attention in recent years. The purpose of this research was to compare complication rates between initial biopsy and rebiopsy in non-small cell lung cancer patients with progressive disease and to assess complication risk factors and clinical results after rebiopsy.

**Methods:**

The study included 113 patients initially diagnosed with non-small cell lung cancer who underwent lung biopsy at initial biopsy and rebiopsy after progression while on epidermal growth factor receptor tyrosine kinase inhibitors (EGFR-TKIs) and/or chemotherapy from January 2018 to December 2021. We compared the incidence of complications between the initial biopsy and rebiopsy and analyzed the predictors factors that influenced complications in patients who underwent rebiopsy.

**Results:**

The successful rate of rebiopsy was 88.5% (100/113). With the exception of two cases where lung adenocarcinoma changed into small cell lung cancer with gefitinib treatment, 98 individuals retained their initial pathological type. The secondary EGFR T790M mutation accounts for 55.6% of acquired resistance. The total number of patients with complications in initial biopsy was 25 (22.1%) and 37 (32.7%) in the rebiopsy. The incidence of pulmonary hemorrhage increased from 7.1% at the initial biopsy to 10.6% at rebiopsy, while the incidence of pneumothorax increased from 14.2% to 20.4%. Compared with the initial biopsy, the incidence of overall complications, parenchymal hemorrhage, and pneumothorax increased by 10.6%, 3.5%, and 6.2%, respectively. In all four evaluations (pneumorrhagia, pneumothorax, pleural reaction, and overall complication), there were no significant differences between the rebiopsy and initial biopsy (all *p* > 0.05). The multivariate logistic regression analysis suggested that male sex (odds ratio [OR] = 5.064, *p* = 0.001), tumor size ≤ 2 cm (OR = 3.367, *p* = 0.013), EGFR-TKIs with chemotherapy (OR = 3.633, *p* =0.023), and transfissural approach (OR = 7.583, *p* = 0.026) were independent risk factors for overall complication after rebiopsy.

**Conclusion:**

Compared with the initial biopsy, the complication rates displayed a slight, but not significant, elevation in rebiopsy. Male sex, tumor size ≤ 2 cm, transfissural approach, and EGFR-TKIs combined with chemotherapy were independent risk factors for rebiopsy complications.

## Introduction

Lung cancer is a significant global health issue as it is responsible for the majority of cancer-related deaths (1). Non-small cell lung cancer (NSCLC) is the primary type of lung cancer and constitutes around 85%–90% of all lung cancer (2). Unfortunately, due to a lack of symptoms and accurate detection at an early stage, a considerable proportion of lung cancer patients are diagnosed at an advanced stage and may only be treated with medications (3). In Chinese patients, 40% of lung cancer patients have epidermal growth factor receptor (EGFR) gene mutations, compared with only 15% in European patients, and these EGFR mutations confer sensitivity to tyrosine kinase inhibitors (TKIs) (4, 5). Indeed, the first-line treatment of advanced NSCLC has evolved from traditional chemotherapy to molecular target and immunotherapy, and the clinical results and use of EGFR-TKIs have significantly improved the overall response rate and progression-free survival of patients with advanced NSCLC (6). However, most patients who initially respond to EGFR-TKIs inevitably develop resistance and then relapse, ultimately leading to death (7). Survival is still seriously affected by cancer metastasis, therapeutic resistance, and relapse (8).

The secondary EGFR T790M mutation, identified in approximately 60% of cases, is the most common acquired resistance mechanism, thereby limiting progression-free survival to approximately 9–14 months (9). Hence, it is critical that the patient clearly understands the resistance mechanism of tissue biopsy in order to make individualized treatment decisions based on tumor-related molecular information. At the time of disease progression, several studies have reported that repeat biopsies were physically invasive, procedurally risky, expensive, and time-consuming, making it more technically difficult to obtain tissue than an initial biopsy and sometimes not feasible (10–12). Rebiopsy can be performed in 50% to 80% of NSCLC patients with progressive disease, and tissue samples for histology analysis were only available in 45% of the patients (12, 13). In most situations, a tissue sample for resistance is the test of first choice because tissue rebiopsy could produce more accurate, more reliable results than a liquid biopsy and is sufficient to predict drug susceptibility, and histopathological changes can be detected in tissue samples (14, 15). According to several previous reports, rebiopsy for patients with non‐small cell lung cancer treatment failure can be performed safely and accurately under CT guidance (10, 16, 17). However, no detailed investigation of the risk factors of NSCLC patients resistant to EGFR-TKIs and/or chemotherapy by CT-guided transthoracic needle biopsy has been reported until now. The purpose of this paper is to assess rebiopsy complication risk factors and clinical outcomes in NSCLC patients with progressive disease. Moreover, we also attempted to compare the different incidences of complications between initial biopsy and rebiopsy.

## Materials and methods

### Patient

**Figure 1:**
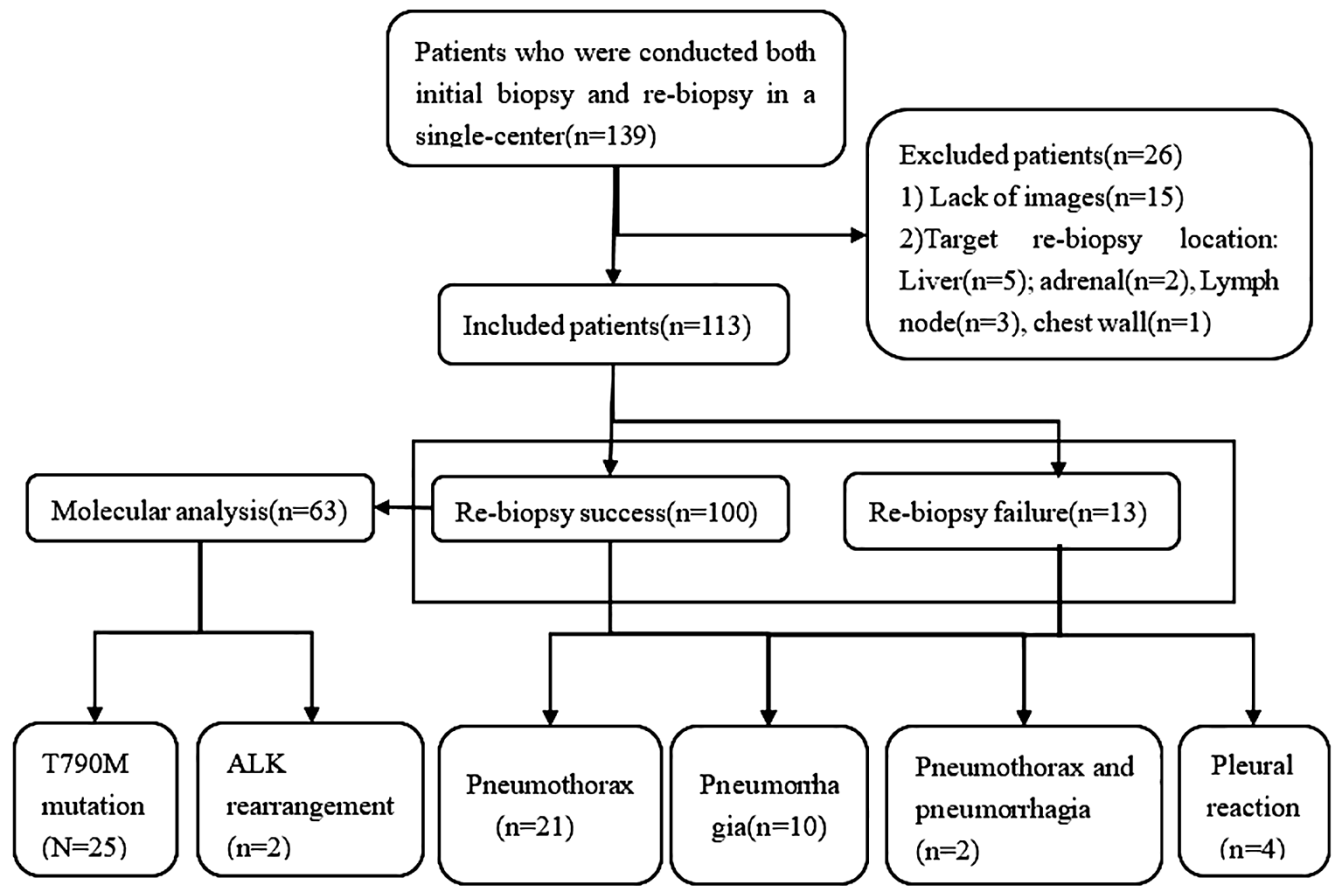
Study flowchart.

This is an observational, single-center, retrospective study approved by the institutional ethical committee. Patients enrolled in the current study were diagnosed pathologically with NSCLC stages IIIB–IV with progressive disease after failure of EGFR-TKIs and/or chemotherapy treatment between January 2019 and December 2022. All included patients underwent CT-guided transthoracic needle biopsy at the initial biopsy and rebiopsy by the same medical team at our hospital. In total, 139 patients were initially screened for inclusion, of whom 26 were excluded, and 113 patients were finally evaluated (**Figure 1**).

The inclusion and exclusion criteria were as follows: (1) patients who had available chest CT scan images on the Picture Archiving and Communication System (PACS); (2) patients with pathologically confirmed NSCLC with progressive disease after failure who had previously received EGFR‐TKIs and/or chemotherapy. We excluded patients who (1) lacked images on the PACS and complete procedural data; (2) the location of initial biopsy and repeat biopsy were extrapulmonary sites.

### Biopsy procedure

All biopsy procedures were performed under local anesthesia by one of five interventional radiologists who had more than 10 years of clinical experience in CT-guided percutaneous lung biopsy. Patients were requested to stop taking any antiplatelet and anticoagulant medications at least 7 days before surgery. All the patients were informed of the procedure, all potential complications, and appropriate treatment and signed the operation informed consent. Prior to biopsy, a chest contrast-enhanced CT scan must be performed in all patients, which helps evaluate the precise location, size of lesions, shape, boundary, and proximity to neighboring organs at risk. Based on the imaging data of the patient, the radiologist determined the appropriate biopsy program.

The principles of biopsy were to choose the safest path to target the lesion, avoiding risk factors such as fissures, blood vessels, emphysema, and bullae. The patient was positioned appropriately according to the lesion location and instructed to breathe calmly. The puncture site was prepared using a sterile technique, and 5–10 ml of 2% lidocaine was used for local infiltration anesthesia to the parietal pleura. The operator subjectively determined the needle insertion angle and the needle insertion distance and then chose the appropriate biopsy method (single-needle or coaxial needle). The single-needle technique, with an 18-gauge biopsy needle, can be advanced into the lesion. When the needle tip was at the edge of the lesion, a tissue sample was obtained. The coaxial needle technique consists of a 17-gauge coaxial trocar together with an 18-gauge automatic biopsy needle. Once the 17-gauge coaxial needle enters the lesion, an automated 18-gauge biopsy needle passes through the lumen of the guide needle. The samples were obtained by core needle biopsy with a 1- or 2-cm needle to ensure safety. Once the specimen was obtained, the operator ensured that there was no blood in the needle channel, inserted the needle core, and withdrew the biopsy needle. It was important to note that there was no on-site pathologist available for immediate evaluation of the biopsy sample during the procedure. Subsequently, the tissue samples were placed in a 10% formalin solution for histopathological examination. Additionally, an immediate postprocedural CT scan was conducted to detect any potential complications.

### Management of complications

Pneumothorax and pulmonary hemorrhage are found mainly on CT scans, while the diagnosis of pleural reaction is based on a series of clinical manifestations. A pneumothorax is the presence of air in the chest cavity. Pulmonary hemorrhage was defined as consolidation or ground glass opacity around the lesion and along the puncture path after biopsy, with or without hemoptysis. Pleural reaction refers to various reactions that occur when the lungs are punctured, such as dizziness, chest tightness, pale complexion, sweating, and even fainting.

The Common Terminology Criteria for Adverse Events (CTCAE) version 5.0 was used to record adverse events. We classified grade 2 or higher complications as more serious complications because grade 1 adverse events are commonly seen based on past experience. Grade 1 pulmonary hemorrhage does not require specialized intervention, but for grade 2 or more severe cases, the patient should be positioned laterally with the puncture point facing downwards, receive prompt intravenous medication to stop bleeding, and undergo continuous monitoring of vital signs. Grade 1 pneumothorax does not require specific treatment. Grade 2 and above require a closed thoracic drainage tube.

### Data collection

All clinical and demographic data were mainly collected from hospital records. The medical records provided information on the patient’s clinical and demographic features, including sex, age, smoking status (yes or no), history of lung surgery (yes or no), cancer stage, Eastern Cooperative Oncology Group (ECOG) performance status, emphysema (yes or no), initial pathology, treatment history (EGFR-TKIs, chemotherapy, and EGFR-TKI combined with chemotherapy), and treatment time.

Additionally, various biopsy-related features were collected from our radiology databases, such as tumor size, tumor location (lobe), target lesion (primary tumor or metastasis), transfissural approach (yes or no), biopsy method (single-needle or coaxial technique), total biopsy time, and pleura-to-target distance.

As a result of reviewing procedure records, medical charts, and follow-up dates, complications relating to the procedure, such as pneumothorax, pneumothorax requiring chest tube placement, pulmonary hemorrhage (with or without hemoptysis), and pleural reaction, were noted.

A successful rebiopsy was defined as obtaining sufficient malignant tissue in biopsy samples. Otherwise, it was defined as failure.

### Statistical analysis

Statistical analysis uses the SPSS 21.0 software. Continuous variables are presented as mean ± standard deviation, while categorical variables are as frequencies (percentages). We counted the incidence of overall complication, pulmonary hemorrhage, pneumothorax, and pleural reaction, respectively, after the initial biopsy and rebiopsy puncture. The McNemar test was used to compare the incidence of complications during the initial biopsy and rebiopsy, and a *p*-value of < 0.05 was considered statistically significant. We then performed comparisons between the complications group and the no complications group to analyze independent risk factors associated with complications. Categorical factors were performed by Pearson Chi‐squared or Fisher exact tests. Continuous data were made using the Mann–Whitney *U* test or independent samples *t*-test.

To identify the risk factors for complications, we performed a binary logistic regression analysis. All covariates with *p* < 0.10 in univariate analysis were included in multivariate analysis. The backward stepwise selection method was used to generate a final reduced model. *p* < 0.05 was considered statistically significant; otherwise, there was no significance.

## Results

### Demographic and clinical characteristics of initial and rebiopsy patients

The population characteristics are shown in **Table 1**. From January 2019 to December 2022, there were 113 patients (mean age: 59.2 years ± 10.5 years, range: 33 to 83 years) who received rebiopsy. There were 54 male (47.8%) patients. Of these patients, 31 (27.4%) were smokers. Adenocarcinomas were found in 101 (89.4%) cases of tumor histology. Regarding treatment history, 37 (32.7%) patients received TKIs, 44 (38.9%) patients received chemotherapy, and 32 (28.3%) patients had received EGFR-TKIs combined with chemotherapy. Primary lesion was biopsied in 66 (58.4%) cases, and the mean tumor size was 3.4 cm ±1.3 cm.

**Table 1:** Demographics and characteristics of 113 patients who received rebiopsy.

Variables	Factors	Number (%)
Sex	Male	54 (47.8)
	Female	59 (52.2)
Mean age (year)		59.2 ± 10.5
Smoking status	Yes	31 (27.4)
History of lung surgery	Yes	18 (15.9)
Stage	IIIB	5 (4.4)
	IV	108 (95.6)
ECOG performance status	0–2	105 (92.9)
	3–4	8 (7.1)
Target lesion	Primary	66 (58.4)
	Metastasis	47 (41.6)
Tumor size (cm)		3.4 ± 1.3
Biopsy site	Upper lobe	58 (51.3)
	Middle lobe	8 (7.1)
	Lower lobe	47 (41.6)
Treatment method	EGFR-TKI	37 (32.7)
	Chemotherapy	44 (38.9)
	TKI plus chemotherapy	32 (28.3)
Initial pathology	Adenocarcinoma	101 (89.4)
	Non-adenocarcinoma	12 (10.6)
Mean treatment time (M)		13.3 ± 5.5

ECOG, Eastern Cooperative Oncology Group; M, months.

### Results of rebiopsy

The successful rate of rebiopsy was 88.5% (100/113). Of the patients, 98 maintained the original pathological type, including adenocarcinoma (*n* = 89), squamous cell carcinoma (*n* = 6), and large cell lung cancer (*n* = 3). We identified two cases of lung adenocarcinoma transformation to small cell lung cancer after treatment with gefitinib. The reason for the failure of rebiopsy was the absence of malignant cells or insufficient malignant cells (*n* = 13). The pathologies of unsuccessful biopsies included lung tissue, fibrocytes, and inflammatory cells. The examination of the driver gene mutation was performed in 45 patients with non-small cell lung cancer who had received the first-generation EGFR-TKIs, and 25 (55.6%) were positive for EGFR T790M mutation.

### Complications of initial biopsy and rebiopsy

Rates of adverse events are shown in **Table 2**, and a comparison of complication probability between initial biopsy and rebiopsy is shown in **Table 3**. The number of patients with complications in the initial biopsy was 25 (22.1%) and 37 (32.7%) in the rebiopsy (two women each had two complications; in total, 39 complications). The main complications of rebiopsy were pulmonary hemorrhage and pneumothorax. Although the incidence of pulmonary hemorrhage and pneumothorax increased from 7.1% to 10.6% and 14.2% to 20.4%, respectively, there were no significant differences in overall complications, pulmonary hemorrhage, pneumothorax, or pleural reaction between initial biopsy and rebiopsy (all *p* > 0.05).

**Table 2:** Rebiopsy outcomes: adverse events.

Parameter	Result (*n* = 113)
Total adverse events	39 (34.5%)
Pneumothorax	
Grade 1	20 (17.7%)
Grade 2	3 (2.7%)
Pulmonary hemorrhage	
Grade 1	2 (1.8%)
Grade 2	9 (7.9%)
Grade 3	1 (0.9%)
Pleural reaction	
Grade 1	4 (3.5%)

Grade 1, clinical management only; grade 2, intervention required; grade 3, hospitalization indicated.

**Table 3:** Comparison of complication probability between initial biopsy and rebiopsy (*n* = 113).

Variables	Complication^b^	Noncomplication	*p*-value
Overall complications			
No	28 (24.8%)	60 (53.1%)	0.097
Yes^a^	9 (8.0%)	16 (14.2%)	
Pneumorrhagia			
No	11 (9.7%)	94 (83.2%)	0.481
Yes^a^	1 (0.9%)	7 (6.2%)	
Pneumothorax			
No	19 (16.8%)	78 (69.0%)	0.281
Yes^a^	4 (3.5%)	12 (10.6%)	
Pleural reaction			
No	4 (3.5%)	106 (93.8%)	1.000
Yes^a^	0 (0%)	3 (2.7%)	

a Complications at initial biopsy.

b Complications at rebiopsy.

**Figure 2:**
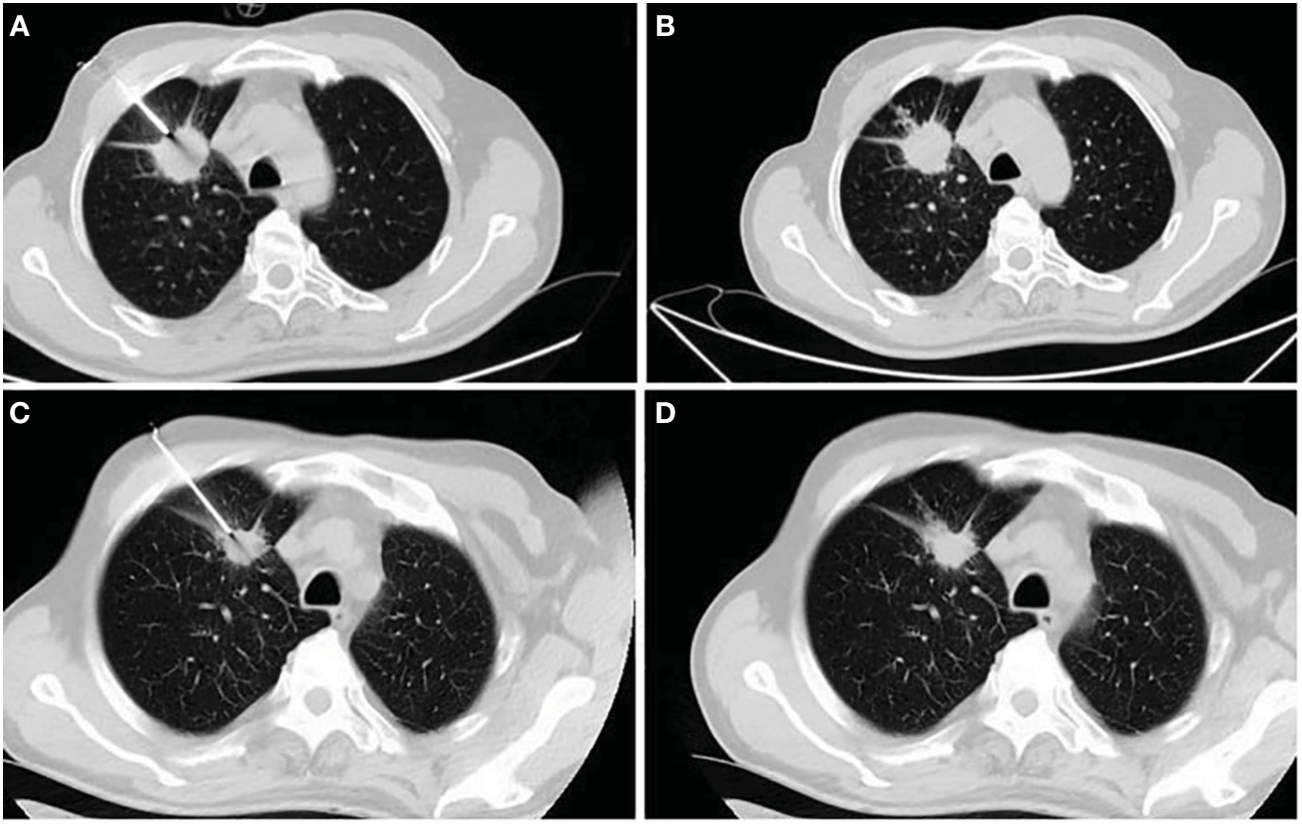
A 62-year-old man receiving his first biopsy **(A, B)** and rebiopsy at age 63 **(C, D)**. **(A)** The needle enters the lesion through coaxial needles at first biopsy. **(B)** CT showed small amounts of bleeding along the needle path. **(C)** Disease progression after 10 months of gefitinib monotherapy and rebiopsy of the primary lesion using a coaxial biopsy needle. **(D)** There were no significant complications after rebiopsy.

Pulmonary hemorrhage was observed in eight patients during the initial biopsy (**Figure 2**), including hemoptysis in four patients. During the rebiopsy, pulmonary hemorrhage occurred in 12 patients, including three cases of hemoptysis. However, only one patient experienced pulmonary hemorrhage in both biopsies. Pneumothorax was observed in 16 patients during the initial biopsy and in 23 patients during the rebiopsy. However, only four patients experienced pneumothorax in both biopsies. Three patients required the placement of a chest tube during the rebiopsy, and X-ray results showed complete absorption of pneumothorax before discharge. Two patients experienced both pneumothorax and pulmonary hemorrhage after the postprocedural biopsy. None of the patients experienced pleural reaction in both biopsies. No serious complications occurred in rebiopsy, such as air embolism, cardiac tamponade, hemodynamic instability, or death.

### Analysis of biopsy-related complications

Statistical modeling can help determine the significant factors that affect the rate of complications for rebiopsy, as shown in **Table 4**. Regarding clinical features, age, smoking status, history of lung surgery, ECOG performance status, emphysema, and treatment time, there were no statistically significant differences (all *p* > 0.10). Male sex (*p* = 0.011) and treatment history (*p* = 0.048) were statistically different (*p* < 0.10). Among the rebiopsy-related factors, tumor size (*p* = 0.083) and transfissural approach (*p* = p0.024) showed *p*-values less than 0.10. Next, the above suggestive factors were included in the binary logistic regression analysis (**Table 5**). The independent risk factors of overall complications were male sex (odds ratio [OR], 5.064; 95% confidence interval [CI]: 1.865, 13.749; *p* = 0.001), tumor size ≤ 2 cm (OR, 3.367; 95% CI: 1.298, 8.735; *p* = 0.013), EGFR-TKIs combined with chemotherapy (OR, 3.633; 95% CI: 1.199, 11.006; *p* = 0.023), and transfissural approach (OR, 7.583; 95% CI: 1.272, 45.209; *p* =0.026).

**Table 4:** The rate of complication for rebiopsy in different factors (*N* = 113).

Variables	Factors	Complication status		*p*-value
		Complication (*n* = 37)	Noncomplication (*n* = 76)	
Age (year)		59.0 ± 9.6	59.2 ± 10.9	0.891
Sex	Male	24 (64.9%)	30 (39.5%)	0.011
	Female	13 (35.1%)	46 (60.5%)	
Smoking status	Yes	13 (35.1%)	18 (23.7%)	0.200
	No	24 (64.9%)	58 (76.3%)	
History of lung surgery	Yes	5 (13.5%)	13 (17.1%)	0.624
	No	32 (86.5%)	63 (82.9%)	
ECOG performance status	0-2	35 (94.6%)	70 (92.1%)	0.926
	3-4	2 (5.4%)	6 (7.9%)	
Emphysema	Yes	2 (5.4%)	5 (6.6%)	1.000
	No	35(94.6%)	71(93.4%)	
Treatment history	TKI	10 (27.0%)	27 (35.5%)	0.048
	Chemotherapy	11 (29.8%)	33 (43.4%)	
	TKI plus chemotherapy	16 (43.2%)	16 (21.1%)	
Treatment time (M)	≤ 15	18(48.6%)	43(56.6%)	0.427
	> 15	19(51.4%)	33(43.4%)	
Target lesion	Primary	21 (56.8%)	45 (59.2%)	0.804
	Metastasis	16 (43.2%)	31 (40.8%)	
Biopsy site	Upper lobe	15 (40.5%)	43 (56.6%)	0.273
	Middle lobe	3 (8.1%)	5 (6.6%)	
	Lower lobe	19 (51.4%)	28 (36.8%)	
Tumor size (cm)	≤ 2	21 (56.8%)	3 0 (39.5%)	0.083
	> 2	16 (43.2%)	46 (60.5%)	
Biopsy method	Single needle	12 (32.4%)	15 (19.7%)	0.138
	Coaxial technique	25 (67.6%)	61 (80.3%)	
Transfissural approach	Yes	6 (16.2%)	2 (2.6%)	0.024
	No	31 (83.8%)	74 (97.4%)	
Pleura-to-target distance (cm)	≤ 3	21 (56.8%)	54 (71.1%)	0.131
	> 3	16 (43.2%)	22 (28.9%)	
Total biopsy time (min)	≤ 10	9 (24.3%)	28 (36.8%)	0.183
	> 10	28 (75.7%)	48 (43.2%)	

**Table 5:** Multivariate analysis of complication (*n* = 113).

Variables	Multivariate analyses	
	OR (95% CI)	*p*-value
Sex (male vs. female)	5.064 (1.865, 13.749)	0.001
Tumor size (≤ 2 cm vs. > 2 cm)	3.367 (1.298, 8.735)	0.013
TKI plus chemotherapy vs. chemotherapy	3.633 (1.199, 11.006)	0.023
Transfissural approach (yes vs. no)	7.583 (1.272, 45.209)	0.026

## Discussion

In this study, we have three main findings: First, CT-guided transthoracic needle rebiopsy was safe and feasible for patients with treatment failure and disease progression. Second, compared with the initial biopsy, the complication rates displayed a slight, but not significant, elevation in rebiopsy. Third, male sex, tumor size ≤ 2 cm, transfissural approach, and EGFR-TKIs combined with chemotherapy were independent risk factors for rebiopsy complications.

Currently, tumor heterogeneity is associated with therapy resistance, malignant progression, and recurrence in cancer. Hence, there is a strong demand for rebiopsy to assess the molecular and pathological characterization of NSCLC patients with progressive disease (18–20). A study of repeat biopsies after EGFR-TKI treatment found that repeat biopsies were technically more difficult than initial biopsies and procedures associated with higher complication rates and failure rates (21).

The rate of surgical resection increased from 1.8% at the initial biopsy to 7.8% at rebiopsy when compared to the biopsy collection method, while the rate of lung biopsy rose obviously from 7.6% to 29.1% (10). This evidence illustrates the technical difficulty in obtaining tissue samples for patients at a repeat biopsy. CT-guided transthoracic needle biopsy is one of the invasive diagnostic methods with highly sensitive and accurate procedures, and complications will inevitably occur during the operation. According to previous reports (22–27), the rate of pulmonary parenchymal hemorrhage ranged from 5% to 26.8%, severe pulmonary hemorrhage or hemoptysis was 0.06% to 7.0%, and the rate of pneumothorax was 12% to 45%, with 2.7% to 6.6% requiring chest tube placement. In the meantime, some interesting prior research has reported that the complication rates in patients with repeat biopsy were similar to those who had a first biopsy (16, 28, 29). Both pulmonary hemorrhage and pneumothorax in rebiopsy were accepted, and pneumothorax was the most common. Also, a meta-analysis (30) showed a slight but non-significant reduction in the pooled complication rate for the rebiopsies vs. the initial biopsies (16.8% vs. 22.2%). Although patients showed disease progression after receiving a previous systemic treatment, the overall complication rate of the biopsy was not statistically significant between the two groups. On the contrary, in our study, all patients underwent repeat biopsies according to disease progression, and the incidences of parenchymal hemorrhage and pneumothorax increased from 7.1% to 10.6% and 14.2% to 20.4%, respectively, from the first biopsy to the second biopsy. For CT-guided biopsy, the overall complication rate at the single center study improved from 22.1% in the initial biopsy to 32.7% in the rebiopsy. In all four evaluations (pneumorrhagia, pneumothorax, pleural reaction, and overall complication), there were no significant differences between the rebiopsy and initial biopsy (all *p* > 0.05). Compared with initial biopsy, the incidence of overall complications, parenchymal hemorrhage, and pneumothorax increased by 10.6%, 3.5%, and 6.2%, respectively, but no serious postoperative complications, including air embolism, severe hemodynamic changes, or death, occurred at the rebiopsy. These findings suggest that rebiopsy is both feasible and safe for NSCLC patients with treatment failure and disease progression.

Male sex, tumor size ≤ 2 cm, transfissural approach, and the use of EGFR-TKIs combined with chemotherapy were independent risk factors for complications even after multivariate analysis. There were several plausible explanations for this result. First, EGFR-TKIs combined with chemotherapy had significant therapeutic benefits, but they may cause or aggravate lung injury, which leads to an increased risk of biopsy-related complications. Previous research has shown that EGFR-TKI-induced lung injury has clinical risk factors, including male sex, smoking status, early treatment initiation after diagnosis, and prior treatment with chemotherapy or radiotherapy (31–33). Likewise, our research also confirmed clinical evidence of a higher risk of complication with concurrent use of EGFR-TKIs combined with chemotherapy relative to chemotherapy alone. Second, the size of the tumor is small, the operation is difficult, and the complication rate is relatively high (34). Notably, accuracy was significantly dependent on tumor size; the accuracy rate of the size of 21–30 mm vs. ≤ 20 mm was significantly higher (97% vs. 85%, *p* < 0.05) (35). Third, a transfissural approach was a significant predictor of pneumothorax, and the risk of parenchymal hemorrhage is also increased when the puncture needle breaks through the pleura more than two times. The more times of repeated punctures of the pleura, the greater the possibility of pneumothorax and parenchymal hemorrhage (36–38).

In addition, there were other factors that may influence the complications of lung rebiopsy. The main factors were as follows: (1) Sampling location: the volume of primary lesion increased, and a large number of tumor angiogenesis and cells were distributed in marginal areas after progression of treatment failure, where tumor cell growth was active, which was the ideal sampling target for rebiopsy, and biopsy increases the risk of complications (39, 40). (2) Patients with underlying disease and cooperation level: stage IIB/IV tumor patients with long-term systemic treatment, especially those men with a history of smoking, have reduced lung basal and vascular fragility, as well as reduced ability to contract after injury, which may increase the challenge of rebiopsies (41, 42). Therefore, accurate measurement of the depth of the needle during a biopsy, minimizing the number of punctures, and avoiding the needle path by passing through bullae, blood vessels, and interlobar fissures can effectively reduce the incidence of complications. In addition, it is necessary to actively communicate with the patient to alleviate their anxiety before the biopsy. The local anesthesia needle should be inserted as close as possible to the parietal pleura to reduce the occurrence of pleural reaction so that the patient can better cooperate during the biopsy.

Several previous studies have shown that the success rate of rebiopsy of advanced lung cancer by transthoracic puncture is about 82%–85% (10, 18, 29). Our slightly higher success rate (88.5%) may be due only to CT-guided biopsy samples compared to that in prior studies. Driven by drug resistance factors, tumor lesions were more advanced than before, the substance components were more complex and diverse, and the lesions in the central region may have a higher incidence of necrosis, resulting in sampling errors in small sample biopsy. For rebiopsy, combined with an enhanced CT scan or PET-CT scan, biopsy metabolically active lesions can improve the detection rate of malignant tumor cells.

In summary, in the real world, rebiopsy is safe after treatment failure in patients with non-small cell lung cancer. The most common complications are pulmonary parenchymal hemorrhage and pneumothorax. Adequate biopsy samples can be used to monitor disease progression, interpret drug resistance mechanisms, and provide a reference for subsequent treatment planning for patients with targeted drug therapy failure or drug resistance (43).

The limitations of our study can be listed as follows: first, a single-center retrospective study fails to record and access some risk factors if those factors were not systematically reported, such as pain. Second, the relatively small sample size fails to reach statistical significance for a certain risk factor. Third, procedure techniques and operators’ experience may also affect the occurrence of complications.

## Data availability statement

The original contributions presented in the study are included in the article/supplementary material. Further inquiries can be directed to the corresponding author.

## Ethics statement

The studies involving humans were approved by Ethics Committee of the Third Affiliated Hospital of Zhengzhou University. The studies were conducted in accordance with the local legislation and institutional requirements. Written informed consent for participation was not required from the participants or the participants’ legal guardians/next of kin because this was a retrospective, single-center study. Written informed consent was obtained from the individual(s) for the publication of any potentially identifiable images or data included in this article.

## Author contributions

YW: Writing – original draft, Writing – review & editing. YZ: Conceptualization, Data curation, Writing – review & editing. NR: Software, Writing – review & editing. FL: Formal analysis, Writing – review & editing. LL: Methodology, Writing – review & editing. ZZ: Data curation, Writing – original draft. MG: Investigation, Writing – original draft. MW: Investigation, Writing – original draft. XZ: Methodology, Writing – original draft.
